# Uncovering BTB and CNC Homology1 (BACH1) as a Novel Cancer Therapeutic Target

**DOI:** 10.3389/fgene.2022.920911

**Published:** 2022-05-16

**Authors:** Zheming Liu, Jing Wang, Huiyong Chen, Zankai Wu, Fuben Liao, Sheng Wang, Ting Zhu

**Affiliations:** ^1^ Cancer Center, Renmin Hospital of Wuhan University, Wuhan, China; ^2^ Reproductive Medicine Centre, Zhongshan Hospital, Fudan University, Shanghai, China; ^3^ Department of Rheumatology, Zhongshan Hospital, Fudan University, Shanghai, China; ^4^ Department of Breast and Thyroid Surgery, Renmin Hospital of Wuhan University, Wuhan, China; ^5^ Institutes of Biomedical Sciences, Fudan University, Shanghai, China; ^6^ Department of Otolaryngology-Head and Neck Surgery, Renmin Hospital of Wuhan University, Wuhan, China

**Keywords:** BTB and CNC homology1 (BACH1), therapeutic target, tumor mutation burden, immune cell infiltration, metabolism

## Abstract

BTB and CNC homology1 (BACH1), working as a transcriptional factor, is demonstrated to function on the regulation of epigenetic modifications by complex regulatory networks. Although BACH1 is reported as an oncogene, the overall analysis of its role remains lacking. In this study, we uncovered the capacity of BACH1 as a new pan-cancer therapeutic target. We found that BACH1 is highly expressed in abundant cancers and correlated with the poor prognosis of most cancers. The mutation sites of BACH1 varied in different cancer types and correlated to patients’ prognoses. The tumor mutation burden (TMB) in four cancer species and up to six tumor infiltrated immune cells had a significant relevance with BACH1. The enrichment analysis showed that the BACH1-associated genes were significantly enriched in the pathways of PD-1/L1 expression, ubiquitin-mediated proteolysis, T cell receptor, Th17 cell differentiation. We then demonstrated that BACH1 is positively correlated with the expression of many candidate genes, incluing SRPK2, GCLM, SLC40A1, and HK2 but negatively correlated with the expression of KEAP1 and GAPDH. Overall, our data shed light on BACH1’s effect on latent utility in cancer targeting therapy.

## Introduction

As the leading cause of mortality, cancer has surpassed cardiovascular and stroke to be the most malignant disease in many countries (GBD 2017 Causes of Death Collaborators, 2018). Chemo-radiotherapy, and surgery are the main traditional regimens for cancer. However, due to the complex mechanism of tumorigenesis, metastasis and recurrence, there are no effective therapeutic strategy to completely cure advanced tumors. With the high-speed development of high throughput sequencing, a mass of data was generated and uploaded to the public databases for exploring novel therapeutic targets, including the transcriptional factors involved in epigenetic modification (Cheng et al., 2021).

BTB and CNC homology 1 (BACH1) is classified as a transcription factor, which is reported to be an oncogene in cancer (Zhang et al., 2018). It is demonstrated that BACH1 is highly expressed in multiple cancers, and involved in metabolism and immune pathways by regulating its target genes. For example, BACH1 expression level is associated with breast cancer recurrence in patients (Yun et al., 2011; Liang et al., 2012). Further studies demonstrate that BACH1 plays its pro-metastatic activity by up-regulating the expression of matrix metalloproteinases (MMP) 1, MMP9, MMP 13, and other pro-metastatic genes (Davudian et al., 2016; Shajari et al., 2018). Meanwhile, through regulating the transcription of electron transport chain (ETC) genes, BACH1 reduces glucose usage in the tricarboxylic cycle in breast cancer cells (Lee et al., 2019), which may partly explain the Warburg effect. Besides, BACH1 also participates in epigenetic modification to promote the progression of cancer. In colorectal cancer and melanoma, BACH1 heterodimerizes with MAF BZIP Transcription Factor G (MAFG), cooperating with chromodomain helicase DNA-binding protein 8 (CHD8) and DNA Methyltransferase 3 Beta (DNMT3B) to inhibit the transcription of many antioncogenes (Fang et al., 2014; Fang et al., 2016). In this paper, by systematically analyzing the data from TCGA database, we present the role of BACH1 as a novel cancer therapeutic target and highlight the latent utility of BACH1 in cancer targeting therapy.

## Methods and Materials

### TIMER2.0 Database Analysis

Tumor Immune Estimation Resource, version 2.0 (TIMER2.0) (http://timer.comp-genomics.org/) is a comprehensive web server providing a robust estimation of immune infiltration and its related tumor molecular and clinical features (Li et al., 2020). We firstly compared the expression levels of BACH1 between normal tissues and tumors across 33 cancer types based on The Cancer Genome Atlas (TCGA) database. Then we studied the correlation of BACH1 expression with tumor purity or the immune cell infiltration including CD8^+^ T, CD4^+^ T cells, B cells, neutrophils, macrophages, and dendritic cells using TIMER2.0. The comparison of immune cell infiltration between BACH1 mutated and non-mutated tumors was displayed in violin plots.

### GEPIA2 Database Analysis

Gene Expression Profile Interactive Analysis, version 2 (GEPIA2) (http://gepia2.cancer-pku.cn/) webserver includes gene expression data from the TCGA database and the Genotype-Tissue Expression (GTEx) database. We used GEPIA2 to analyze BACH1 expression in several cancer types that were absent in TIMER2. 0.(Tang et al., 2019). GEPIA2 was also used to compare the overall survival (OS) of cancer patients separated by the optimal cutoffs of BACH1 expression levels and to generate Kaplan-Meier survival curves for 33 different types of cancer. *p*-values for log-rank tests were calculated to evaluate the significance between “high” and “low” groups of BACH1 expression. Survival plots showed the effect of BACH1 expression level on prognosis.

### CPTAC Dataset Analysis

The Clinical Proteomic Tumor Analysis Consortium (CPTAC) dataset provides proteomic expression profiles of cancer biospecimens, including breast cancer, ovarian cancer, colon cancer, clear cell renal cell carcinoma (KIRC), uterine corpus endometrial carcinoma (UCEC), lung adenocarcinoma (LUAD), and pediatric brain cancer. We analyzed the differential expression of BACH1 protein between primary tumors and normal tissues based on the UALCAN portal (http://ualcan.path.uab.edu/), which contains total protein and phosphoprotein data from the CPTAC dataset (Chandrashekar et al., 2017). We also compared the expression of BACH1 phosphoproteins with phosphorylation at the S196, S388, S445, T410, S417, and S443 sites in tumors and normal tissues.

### Mutation Profiles

We investigated the mutation landscape and copy number alteration (CNA) of BACH1 in pan-cancer using the cBioPortal for Cancer Genomics (http://www.cbioportal.org). And then, we obtained the survival curves of different types of cancer patients. The somatic mutation datasets of 33 cancer types were downloaded from the TCGA database (https://gdc.cancer.gov). The mutated genes and the classification of mutation types are visualized by R package “Maftools” (Mayakonda et al., 2018). Tumor Mutation Burden (TMB) was calculated by the following formula: TMB = the number of non-synonymous mutations/exon length (38 million). The non-synonymous mutations contain Missense mutation, Nonsense mutation, Frameshift deletion, Splice site mutation, Frameshift insertion, In-frame deletion, Translation start site, Nonstop mutation, and In-frame insertion.

### Functional Enrichment Analysis

We downloaded a list including the genes associated with BACH1 from Ualcan (Supplementary Table S1) and the genes with Pearson correlation coefficient >0.6 were selected for further study. Gene Ontology (GO) functional enrichment analysis and Kyoto Encyclopedia of Genes and Genomes (KEGG) pathway enrichment were performed using the R package “cluster profile”. Bubble plots were drawn using the package “enrichplot” and “ggplot2”. and chord charts were drawn using the GO chord function in the package GOplot. FDR<0.05 was set as the threshold.

## Results

### Pan-Cancer Expression Landscape of BACH1

**FIGURE 1:**
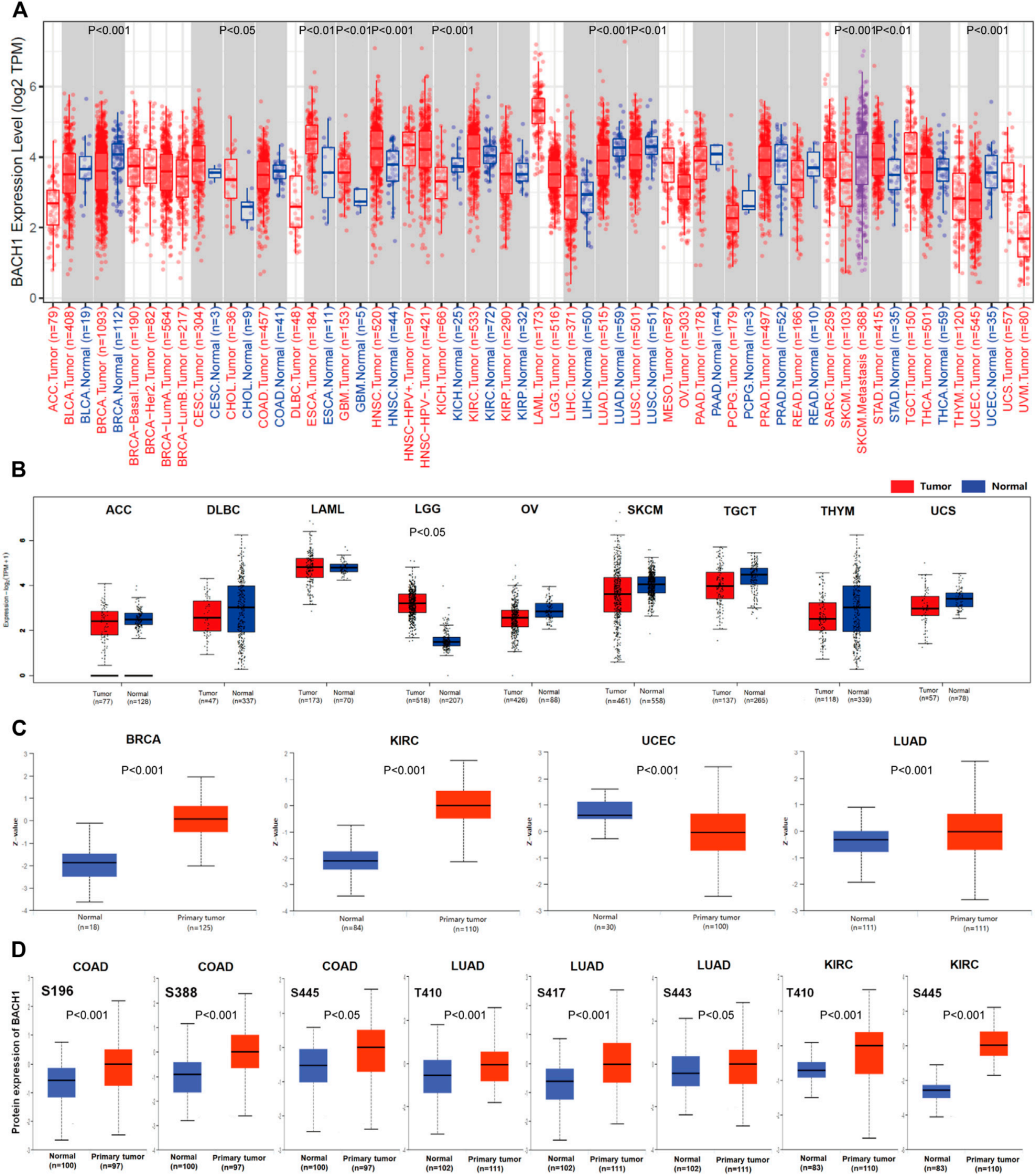
Pan-cancer expression landscape of BACH1. **(A)** The expression of BACH1 in 33 cancer tissues from the TCGA database **(B)** The expression of BACH1 in GTEx and TCGA database. **(C)** The expression level of BACH1 in primary cancer and normal tissue in the CPTAC database **(D)** The expression level of BACH1 phosphosites in different cancer tissues.

BACH1’s expression significantly varies in different cancer types. To explore the difference of BACH1 at the level of pan-cancer, we use TIMER2.0 to compare the expression levels of BACH1 in cancer tissues and the corresponding normal tissues of 33 cancer. The expression of BACH1 increases in Breast invasive carcinoma (BRCA *p* < 0.001), Kidney Chromophobe (KICH *p* < 0.001), Lung adenocarcinoma (LUAD, *p* < 0.0010), Lung squamous cell carcinoma (LUSC, *p* < 0.01), Uterine Corpus Endometrial Carcinoma (UCEC, *p* < 0.001) tumor tissue, and a decrease in Cholangiocarcinoma (CHOL, *p* < 0.050, Esophageal carcinoma (ESCA, *p* < 0.01), Glioblastoma multiforme (GBM, *p* < 0.010, Head and Neck squamous cell carcinoma (HNSC, *p* < 0.001), Stomach adenocarcinoma (STAD, *p* < 0.01) tumor tissues. In addition, BACH1 in the Skin Cutaneous Melanoma (SKCM) metastatic tumor tissue was significantly higher than that in the SKCM tumor tissue (*p* < 0.001) (Figure 1A). To further analyze BACH1 expression in some cancer types lacking corresponding normal tissue data in the TCGA database, we combined the normal tissue data from GTEx and TCGA databases. BACH1 showed upregulated expression in Brain Lower Grade Glioma (BLGG), but there were no significant differences in tumor tissues like Adrenocortical carcinoma (ACC), Diffuse Large B-cell Lymphoma (DLBC), Acute Myeloid Leukemia (LAML), Ovarian serous cystadenocarcinoma (OV), Skin Cutaneous Melanoma (SKCM), Testicular Germ Cell Tumors (TGCT), Thymoma (THYM), Uterine Carcinosarcoma (UCS) compared to their corresponding normal tissues (Figure 1B).

In addition, we use the CPTAC database to analyze differences between BACH1 expression level in primary cancer tissues and normal tissues. Compared with normal tissues, the expression of BACH1 protein is upregulated in primary cancer tissues of breast cancer, KIRC, UCEC and LUAD (Figure 1C). The phosphorylation of BACH1 is highly expressed in primary cancer tissues of colon cancer (phosphosites S196, S388, S445), LUAD (phosphosites T410, S443) (Figure 1D).

### Pan-Cancer Analysis of the Association Between BACH1 Expression and Prognosis

**FIGURE 2:**
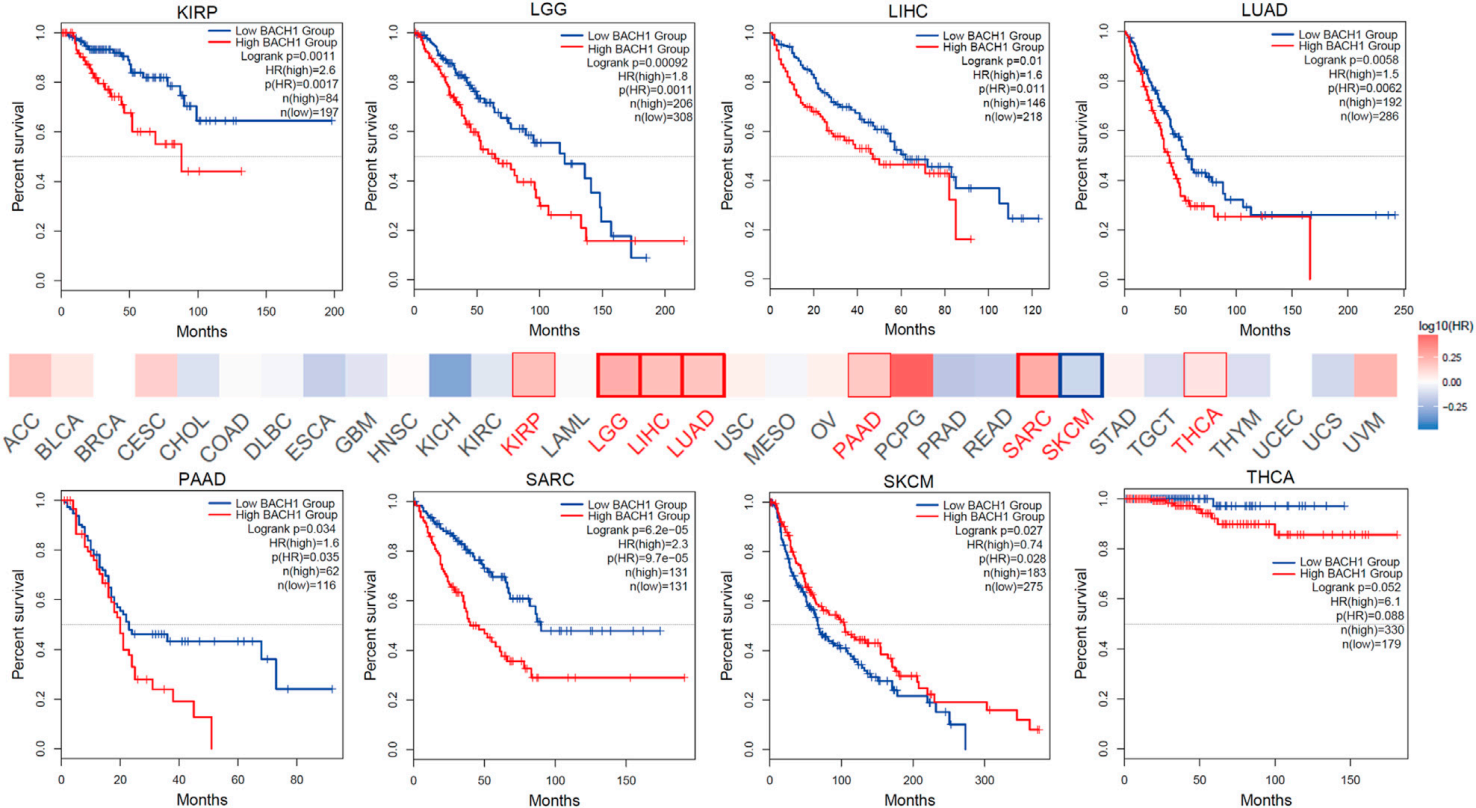
Expression of BACH1 and its association with prognosis in different cancers.

Aiming to reveal the relationship between BACH1 expression and prognosis of tumor patients, the patients were divided into high-expression groups and low-expression groups based on the BACH1 expression level. Patients with low BACH1 expression in groups like KIRP (Kidney renal papillary cell carcinoma, *p* = 0.0011), LGG (lower grade glioblastoma, *p* < 0.001), LIHC (Liver hepatocellular carcinoma, *p* = 0.01), PAAD (Pancreatic adenocarcinoma, *p* = 0.034), SARC (Sarcoma, *p* < 0.001), THCA (Thyroid carcinoma, *p* = 0.052) had a better prognosis than that with high BACH1 expression. While in SKCM (*p* = 0.027) patients, BACH1 high expression patients have a better prognosis (Figure 2).

### Pan-Cancer Mutation Landscape of BACH1

**FIGURE 3:**
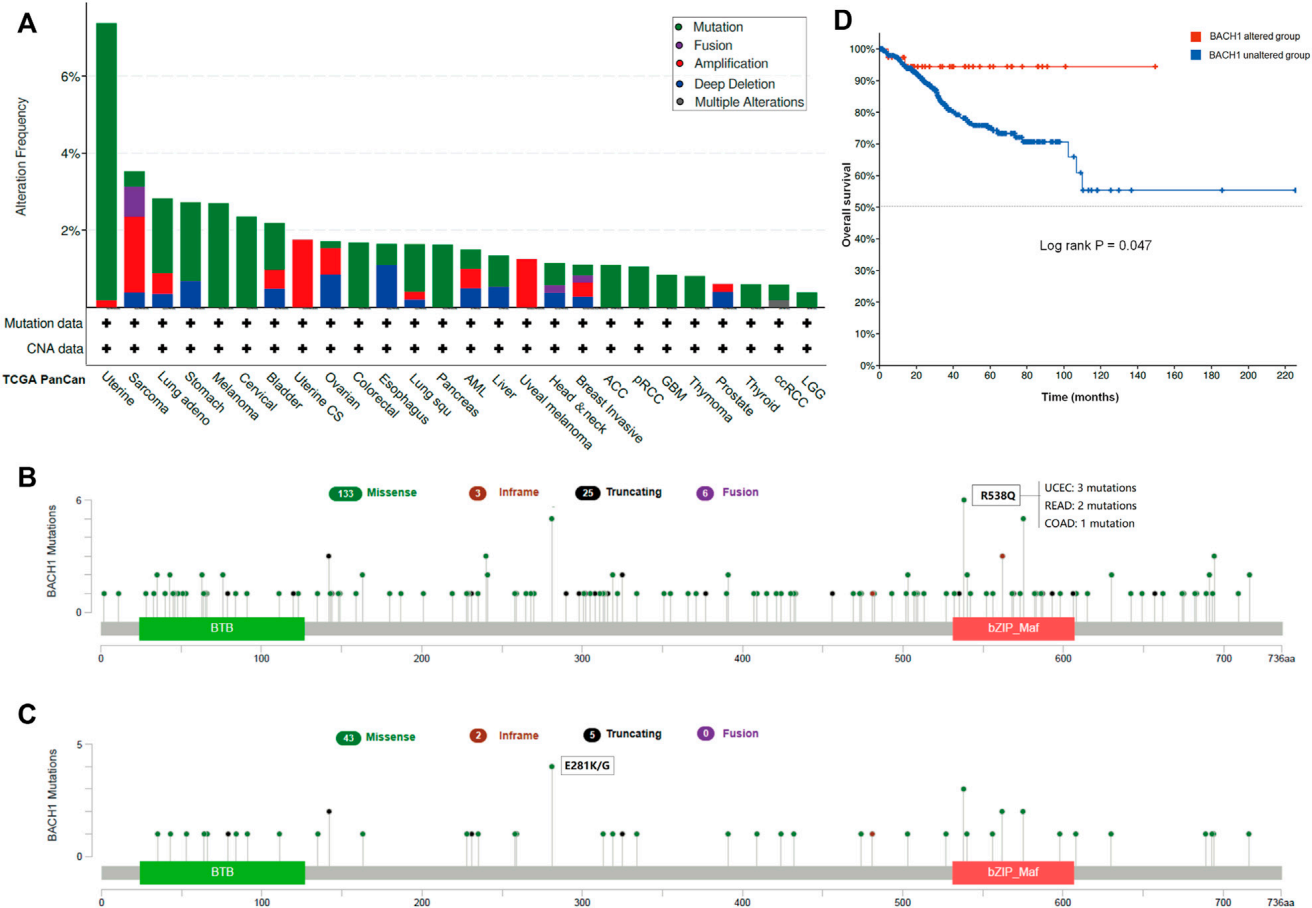
Pan-cancer Mutation Landscape of BACH1. **(A)** Distribution of BACH1 mutation types in different cancers **(B)** Common mutation sites of BACH1 in different cancers. **(C)** Common mutation sites of BACH1 in UCEC **(D)** The overall survival of UCEC patients with BACH1 mutation.

Gene mutation is commonly occurred in cancer cells. We analyzed the BACH1 mutation frequency and mutation sites of patients in different cancer types using cBioPortal. The result showed that UCEC patients were burdened with the highest BACH1 mutation frequency (Figure 3A). BACH1 has 167 mutation sites (including 133 missense, 3 subunits, 25 truncation and 6 fusion mutations) in the protein structure of 0–736 amino acids, of which R538Q and E281 K/G are the two most common mutation sites). (Figures 3B,C). There are 3 cases of R538Q with BACH1 mutation, 2 cases of READ (Rectum adenocarcinoma), and 1 case of COAD (Colon adenocarcinoma) (Figure 3B). Subsequently, we analyzed the prognosis of BACH1 mutations in different cancer types. The total survival of UCEC patients with BACH1 mutation is longer than that of the wild-type group (*p* = 0.047) (Figure 3D).

**FIGURE 4:**
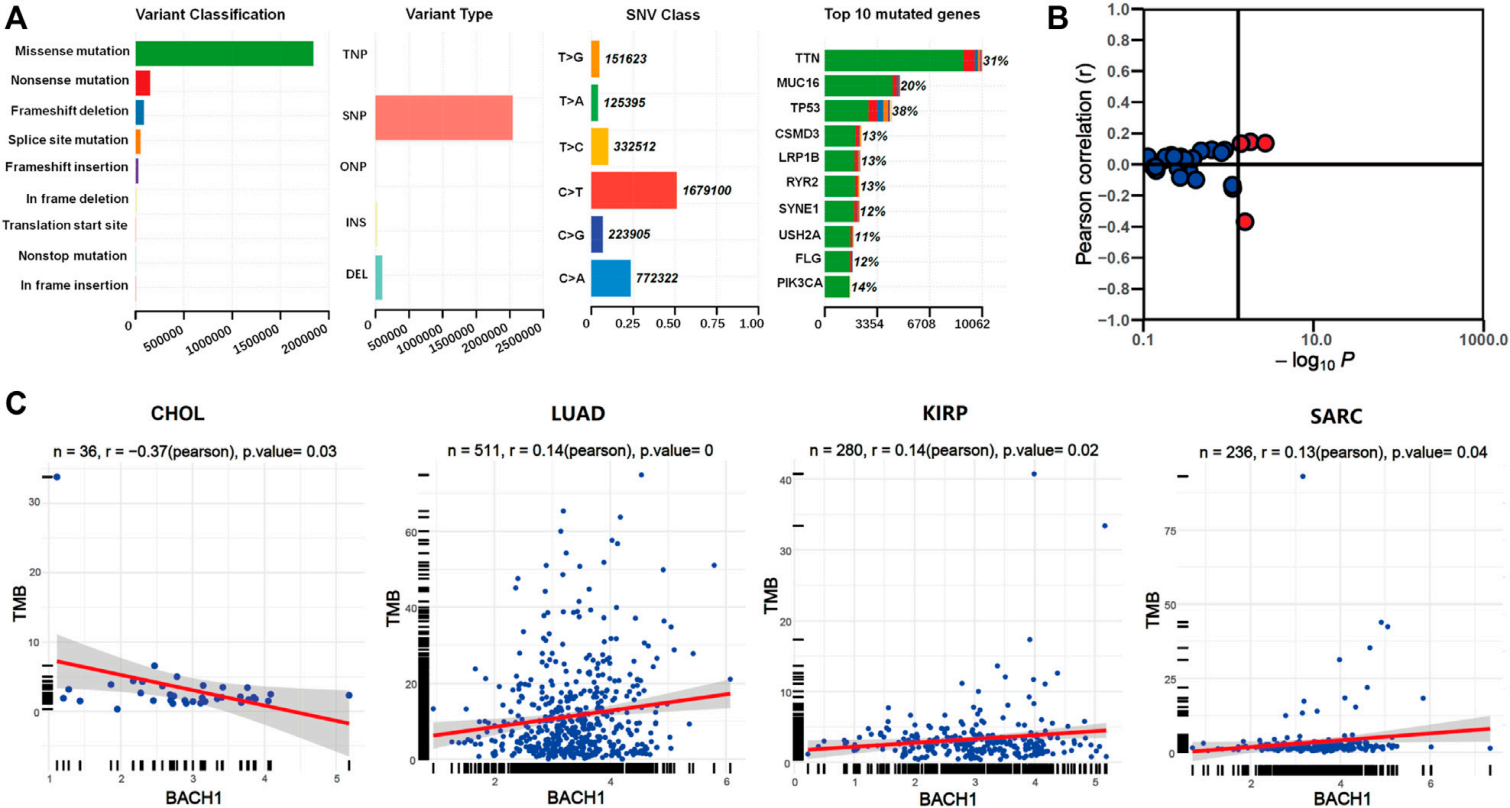
The relationship between BACH1 expression and somatic mutation. **(A)** The somatic mutation data of 33 cancer patients from the TCGA database **(B)** The relationship between BACH1 and TMB **(C)** Four cancer types in which BACH1 is associated with TMB.

We downloaded the somatic mutation data of 33 cancer patients from the TCGA database, and the visualization was carried out using the Maftools R package. The first three most common variant classifications are missence mutation, nonsense mutations, and frameshift deletion. Single nucleotide polymorphism (SNP) is the most common variant type, followed by is deletion mutation. In single-nucleotide variation (SNVs) classes, C > T and C > A are the two most common variant SNV types. The top 10 genes with the highest mutation frequency among pan-cancer patients are: TTN (31%), MUC16 (20%), TP53 (18%), CSMD3 (13%), LRP1B (13%), RYR2 (13%), SYNE1 (12%), USH2A (11%), FLG (12%), and PIK3CA (14%) (Figure 4A).

We calculated the tumor mutation burden (TMB) of pan-cancer samples in the TCGA database and explored its relevance to BACH1 expression. The BACH1 expression and TMB was significantly correlated in 4 cancer types. (Figure 4B). Among them, the TMB is negatively related to the BACH1 expression in CHOL samples (*p* = 0.03), while positively correlated in LUAD (*p* = 0), KIRP (*p* = 0.02) and SARC (*p* = 0.04) (Figure 4C).

### Correlation of BACH1 With Immune Cell Infiltration

Previous reports showed the involvement of BACH1 in the immune system (Zhang et al., 2018). To access the function of BACH1 on tumor immunity, TIMER algorithm was used to study the potential relationship between BACH1 expression in different cancer types and tumor-infiltrated immune cells. We found that the expression of BACH1 in most cancer types is positively related to the level of TILs infiltration. In breast cancer (BRCA) tissues, the BACH1 expression was positively related to the infiltrating levels of CD8/CD4+ T cells, neutrophils, macrophages, and myeloid dendritic cells; but negatively related to that of B cells. The expression of BACH1 in Head and Neck squamous cell carcinoma (HNSC) has positive correlation to the infiltration of CD8^+^ T cells, B cells, neutrophils, macrophages, and myeloid dendritic cells, but no relative infiltration of CD4^+^ T cells. A similar trend of cell infiltration was observed in Kidney Chromophobe (KICH). In Kidney Renal Clear Cell Carcinoma (KIRC), the BACH1 expression is positively related to the infiltration of CD4^+^ T cells, neutrophils, macrophages, and myeloid dendritic cells, but negatively related to B cells. Except for the negative correlation to the infiltration of CD8^+^ T cells in low grade glioblastoma (LGG), the BACH1 expression level is positively related to the infiltration of the other five kinds of immune cells. BACH1’s expression in Liver hepatocellular carcinoma (LIHC) tumor tissue is positively correlated with all six kinds of immune cells.

**FIGURE 5:**
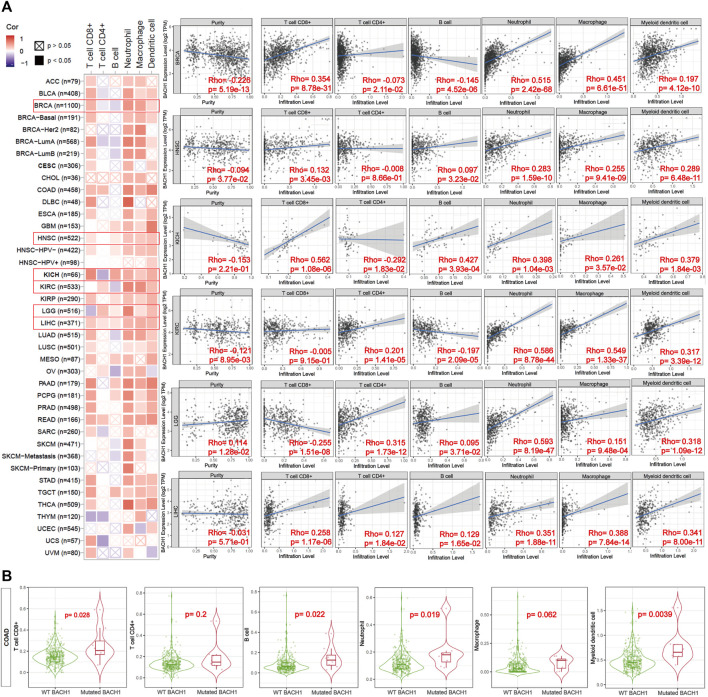
Correlation of the expression or mutation of BACH1 with immune infiltration. **(A)** Correlation of BACH1 Expression with Immune Infiltration **(B)** Correlation of BACH1 mutation with Immune Infiltration.

Tumor purity reflects the proportion of tumor cells in TME (Rhee et al., 2018). The lower the purity of the tumor, the higher the proportion of infiltrated immune cells. The expression of BACH1 in BRCA and HNSC tumor tissue is negatively related to the purity of the tumor, while BACH1 in LGG is positively related to that of the tumor (Figure 5A).

BACH1 mutations also impact the infiltration of immune cells. BACH1 mutated COAD has more obvious correlation with infiltrated CD8^+^ T cells (*p* = 0.028), B cells (*p* = 0.022), neutrophils (*p* = 0.019), and myeloid dendritic cells (*p* = 0.0039) than that of BACH1 wild-type group. Nonetheless, the infiltrating degree of CD4^+^ T cells and macrophages has no statistical significance (Figure 5B).

### Functional Enrichment Analysis

**FIGURE 6:**
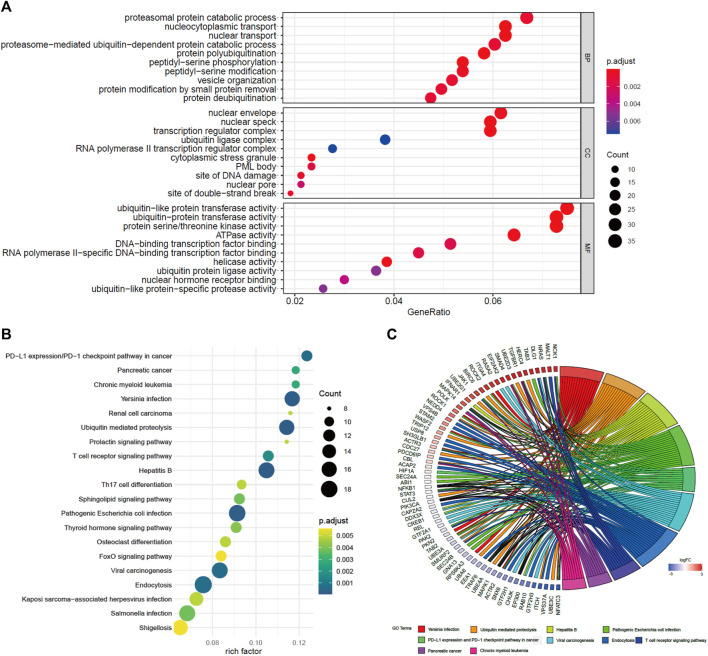
GO and KEGG pathway enrichment analysis of BACH1. **(A)** GO pathway enrichment analysis of BACH1 **(B)**-**(C)** KEGG pathway enrichment analysis of BACH1.

To study the potential functions of BACH1, we screened the genes related to BACH1 expression for functional enrichment analysis. The results of the GO pathway enrichment analysis (Rhee et al., 2008) showed that the top 10 BACH1 expression relevant genes are mainly involved in biological processes such as proteasomal protein catabolic process, nucleocytoplasmic transport, nuclear envelope, and nuclear transport (BP); Participating cell composition (CC) includes the nuclear envelope, nuclear speck, and transcription regulator complex, etc. The major molecular function (MF) includes protein transferase activity, protein serine/threonine kinase activity, and ATPase activity (Figure 6A). In addition, the KEGG pathway enrichment analysis (Kanehisa and Sato, 2020) showed that BACH1 expression-related genes are mainly involved in PD-1/L1 expression, ubiquitin-mediated proteolysis, T cell receptor, Th17 cell differentiation, and endocytosis and other signal channels (Figures 6B,C).

**FIGURE 7:**
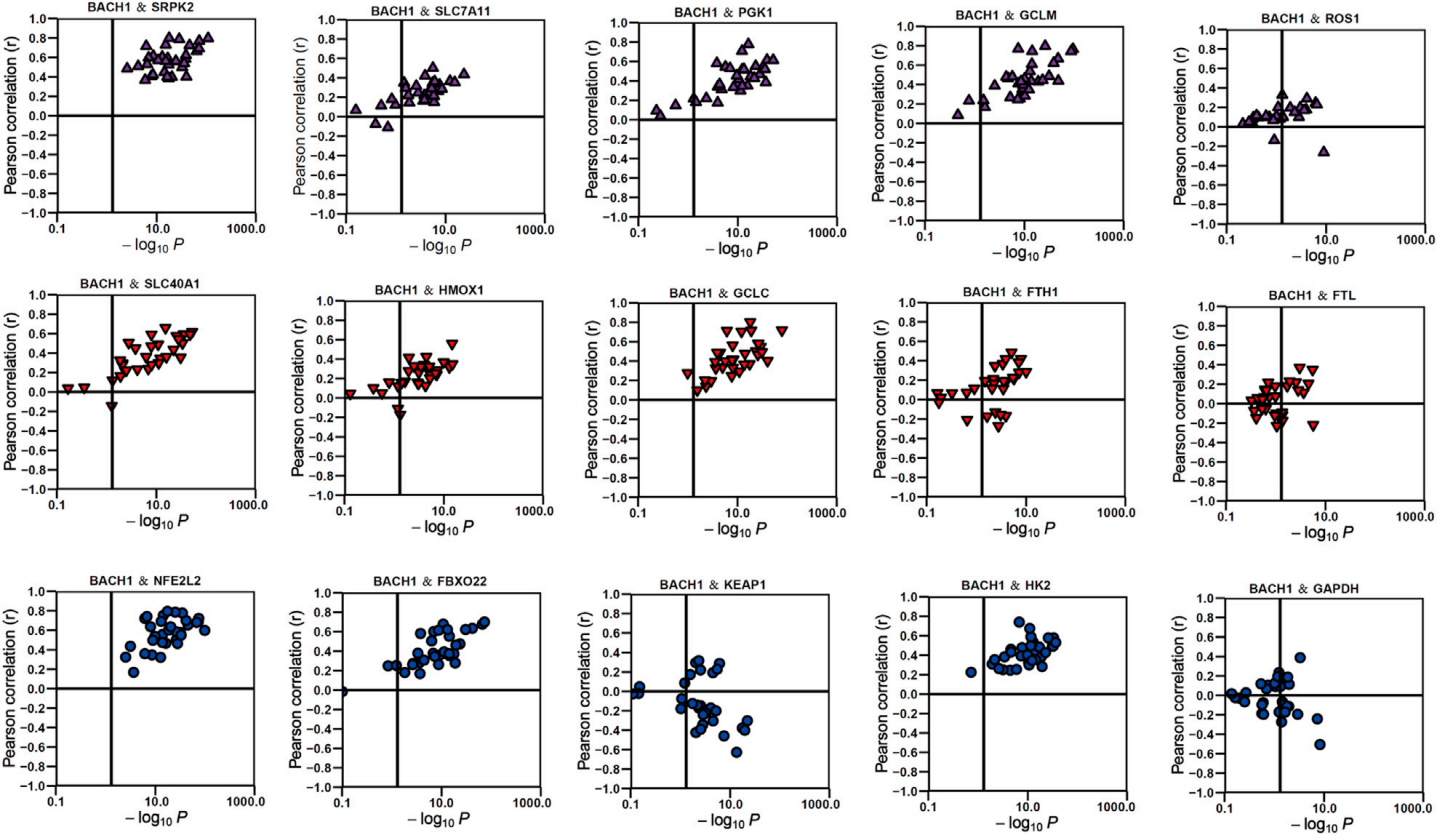
The correlation of BACH1 and metabolism-related genes.

There are many researches that have reported the vital role of BACH1 in metabolism. Our KEGG analysis also showed that BACH1 was correlated with metabolic diseases like diabetes and athrosclerosis. We then analyzed its effect on physiological metabolisms like redox, glycolysis, amino acid metabolism, heme metabolism, and ferroptosis. The data showed that BACH1 is positively correlated with the expression of many related genes such as SRPK2, GCLM, SLC40A1, and HK2 but negatively correlated with the expression of KEAP1 and GAPDH (Figure 7).

## Discussion

Through systematic analysis of BACH1 expression and function in different cancer types, we demonstrated the profound impact of BACH1 on tumor physiology and overall survival. Until now, our research is the first to thoroughly elucidate the correlation between BACH1 expression and OS or physiology in cancer patients at pan-cancer level.

As we presented above, BACH1 is highly expressed in most tumors. The higher BACH1 expressed, the worse overall survival of cancer patients would have. Nonetheless, SKCM is the only cancer whose expression negatively correlates with the OS of patients. The LUAD, KIRP, and SARC patients with mutated BACH1 commonly positively correlate with TMB. The mutated BACH1 is positively associated with the prognosis. Our data analysis also showed the correlation of BACH1 with tumor infiltrated immune cells, including lymphoid cells and myeloid cells, for example, CD8+/4 + T cells, B cells, neutrophils, macrophages, and so on. GO pathway enrichment analysis showed that BACH1 participated in cellular physiological metabolic and proliferation. We also found that BACH1 is relevant to PD1/L1, the TCR signaling pathway, Th17, and differentiation in some cancer types. Our KEGG and Pearson correlation analysis hints that BACH1 orchestrate a net of metabolic processes involved in glycolysis, ferroptosis, endocytosis, etc.

High expression of BACH1 was correlated with high TMB, which may be a new biomarker of the prognosis of immunotherapy. Our results and other studies reported the effect of BACH1 on macrophages, which modulates the macrophage fraction (Igarashi et al., 2017) and may modifies the TME in pathophysiologic. A recent study by Reinfeld et al., reported that M2-macrophage is identified as the tumor-associated macrophages (TAMs) and consume the bulk of the glucose in TME (Reinfeld et al., 2021). Consistent with that study, other researchers found that BACH1 modulates ferroptosis in cooperation with haem (Nishizawa et al., 2020). As we all known, the iron level in blood can affect the differentiation of macrophages (Igarashi et al., 2017). Whether BACH1 participates in this process and what role it may play requires further exploration.

According to Lignitto et al., the combination of BACH1 with a ubiquitin ligase, Fbxo22, can eventually induce the degradation of BACH1. (Lignitto et al., 2019). They also explained that Nrf2 promotes the metastasis of human lung cancer by producing the heme oxygenase, Ho1. Another research conducted by Wiel et al. (Wiel et al., 2019) uncovered that BACH1 can initiate a glucose dependent metastatic pathway by activating the important enzymes, Hexokinase2 (HK2) and GAPDH. It speeds up the whole process of glucose metabolism including glucose uptake and lactic acid secretion. And the increased lactic acid may be a nutrient for the regulatory T cells in turn and thus facilitate immunotherapy resistance (Watson et al., 2021). These results are also consistent with our data analysis that BACH1 is highly correlated with the PD1/L1 pathway. But its specific function in immunotherapy resistance still requires further study. Liang et al.(Liang et al., 2012) reported that BACH1 significantly contributes to the bone metastasis of breast cancer by regulating some metastasis-associated genes. For example, CXCR4, which may accelerate progenitors’ homing to the bone marrow and maintain homeostasis of hematopoietic stem cells (HSCs) through CXCL12-CXCR4 axis.

This study highlights the essential role of BACH1 through in-depth analysis of the relationship between BACH1 and OS, cancer metabolism, TMB and immune cell infiltration. The result presented the potential of BACH1 to be a reliable biomarker for the prognosis of pan-cancer patients and a new target for cancer treatment. However, as a retrospective study, the predictive value of BACH1 in cancer prognosis needs to be validated by both basic and clinical studies.

## Data Availability

The original contributions presented in the study are included in the article/Supplementary Material, further inquiries can be directed to the corresponding author.

## References

[B1] Chandrashekar D. S. Bashel B. Balasubramanya S. A. H. Creighton C. J. Ponce-Rodriguez I. Chakravarthi B. V. S. K. (2017). UALCAN: A Portal for Facilitating Tumor Subgroup Gene Expression and Survival Analyses. Neoplasia 19 (8), 649–658. 10.1016/j.neo.2017.05.002 28732212 PMC5516091

[B2] Cheng X. Wang X. Nie K. Cheng L. Zhang Z. Hu Y. (2021). Systematic Pan-Cancer Analysis Identifies TREM2 as an Immunological and Prognostic Biomarker. Front. Immunol. 12, 646523. 10.3389/fimmu.2021.646523 33679809 PMC7925850

[B3] Davudian S. Shajari N. Kazemi T. Mansoori B. Salehi S. Mohammadi A. (2016). BACH1 Silencing by siRNA Inhibits Migration of HT-29 Colon Cancer Cells through Reduction of Metastasis-Related Genes. Biomed. Pharmacother. 84, 191–198. 10.1016/j.biopha.2016.09.021 27657827

[B4] Fang M. Hutchinson L. Deng A. Green M. R. (2016). Common BRAF(V600E)-directed Pathway Mediates Widespread Epigenetic Silencing in Colorectal Cancer and Melanoma. Proc. Natl. Acad. Sci. U.S.A. 113 (5), 1250–1255. 10.1073/pnas.1525619113 26787892 PMC4747705

[B5] Fang M. Ou J. Hutchinson L. Green M. R. (2014). The BRAF Oncoprotein Functions through the Transcriptional Repressor MAFG to Mediate the CpG Island Methylator Phenotype. Mol. Cell 55 (6), 904–915. 10.1016/j.molcel.2014.08.010 25219500 PMC4170521

[B6] GBD 2017 Causes of Death Collaborators (2018). Global, Regional, and National Age-sex-specific Mortality for 282 Causes of Death in 195 Countries and Territories, 1980-2017: a Systematic Analysis for the Global Burden of Disease Study 2017. Lancet 392 (10159), 1736–1788. 10.1016/s0140-6736(18)32203-7 30496103 PMC6227606

[B7] Igarashi K. Kurosaki T. Roychoudhuri R. (2017). BACH Transcription Factors in Innate and Adaptive Immunity. Nat. Rev. Immunol. 17 (7), 437–450. 10.1038/nri.2017.26 28461702

[B8] Kanehisa M. Sato Y. (2020). KEGG Mapper for Inferring Cellular Functions from Protein Sequences. Protein Sci. 29 (1), 28–35. 10.1002/pro.3711 31423653 PMC6933857

[B9] Lee J. Yesilkanal A. E. Wynne J. P. Frankenberger C. Liu J. Yan J. (2019). Effective Breast Cancer Combination Therapy Targeting BACH1 and Mitochondrial Metabolism. Nature 568 (7751), 254–258. 10.1038/s41586-019-1005-x 30842661 PMC6698916

[B10] Li T. Fu J. Zeng Z. Cohen D. Li J. Chen Q. (2020). TIMER2.0 for Analysis of Tumor-Infiltrating Immune Cells. Nucleic Acids Res. 48 (W1), W509–w514. 10.1093/nar/gkaa407 32442275 PMC7319575

[B11] Liang Y. Wu H. Lei R. Chong R. A. Wei Y. Lu X. (2012). Transcriptional Network Analysis Identifies BACH1 as a Master Regulator of Breast Cancer Bone Metastasis. J. Biol. Chem. 287 (40), 33533–33544. 10.1074/jbc.M112.392332 22875853 PMC3460454

[B12] Lignitto L. LeBoeuf S. E. Homer H. Jiang S. Askenazi M. Karakousi T. R. (2019). Nrf2 Activation Promotes Lung Cancer Metastasis by Inhibiting the Degradation of Bach1. Cell 178 (2)**,** 316–329. 10.1016/j.cell.2019.06.003 31257023 PMC6625921

[B13] Mayakonda A. Lin D.-C. Assenov Y. Plass C. Koeffler H. P. (2018). Maftools: Efficient and Comprehensive Analysis of Somatic Variants in Cancer. Genome Res. 28 (11), 1747–1756. 10.1101/gr.239244.118 30341162 PMC6211645

[B14] Nishizawa H. Matsumoto M. Shindo T. Saigusa D. Kato H. Suzuki K. (2020). Ferroptosis Is Controlled by the Coordinated Transcriptional Regulation of Glutathione and Labile Iron Metabolism by the Transcription Factor BACH1. J. Biol. Chem. 295 (1), 69–82. 10.1074/jbc.RA119.009548 31740582 PMC6952604

[B15] Reinfeld B. I. Madden M. Z. Wolf M. M. Chytil A. Bader J. E. Patterson A. R. (2021). Cell-programmed Nutrient Partitioning in the Tumour Microenvironment. Nature 593 (7858), 282–288. 10.1038/s41586-021-03442-1 33828302 PMC8122068

[B16] Rhee J.-K. Jung Y. C. Kim K. R. Yoo J. Kim J. Lee Y.-J. (2018). Impact of Tumor Purity on Immune Gene Expression and Clustering Analyses across Multiple Cancer Types. Cancer Immunol. Res. 6 (1), 87–97. 10.1158/2326-6066.Cir-17-0201 29141981

[B17] Shajari N. Davudian S. Kazemi T. Mansoori B. Salehi S. Khaze Shahgoli V. (2018). Silencing of BACH1 Inhibits Invasion and Migration of Prostate Cancer Cells by Altering Metastasis-Related Gene Expression. Artif. Cells, Nanomedicine, Biotechnol. 46 (7), 1495–1504. 10.1080/21691401.2017.1374284 28889753

[B18] Tang Z. Kang B. Li C. Chen T. Zhang Z. (2019). GEPIA2: an Enhanced Web Server for Large-Scale Expression Profiling and Interactive Analysis. Nucleic Acids Res. 47 (W1), W556–W560. 10.1093/nar/gkz430 31114875 PMC6602440

[B19] Watson M. J. Vignali P. D. A. Mullett S. J. Overacre-Delgoffe A. E. Peralta R. M. Grebinoski S. (2021). Metabolic Support of Tumour-Infiltrating Regulatory T Cells by Lactic Acid. Nature 591 (7851), 645–651. 10.1038/s41586-020-03045-2 33589820 PMC7990682

[B20] Wiel C. Le Gal K. Ibrahim M. X. Jahangir C. A. Kashif M. Yao H. (2019). BACH1 Stabilization by Antioxidants Stimulates Lung Cancer Metastasis. Cell 178 (2)**,** 330–345. 10.1016/j.cell.2019.06.005 31257027

[B21] Yon Rhee S. Wood V. Dolinski K. Draghici S. (2008). Use and Misuse of the Gene Ontology Annotations. Nat. Rev. Genet. 9 (7), 509–515. 10.1038/nrg2363 18475267

[B22] Yun J. Frankenberger C. A. Kuo W.-L. Boelens M. C. Eves E. M. Cheng N. (2011). Signalling Pathway for RKIP and Let-7 Regulates and Predicts Metastatic Breast Cancer. Embo J. 30 (21), 4500–4514. 10.1038/emboj.2011.312 21873975 PMC3230370

[B23] Zhang X. Guo J. Wei X. Niu C. Jia M. Li Q. (2018). Bach1: Function, Regulation, and Involvement in Disease. Oxidative Med. Cell. Longev. 2018, 1–8. 10.1155/2018/1347969 PMC6189649 30370001

